# Probiotic Supplements Beneficially Affect Tryptophan–Kynurenine Metabolism and Reduce the Incidence of Upper Respiratory Tract Infections in Trained Athletes: A Randomized, Double-Blinded, Placebo-Controlled Trial

**DOI:** 10.3390/nu8110752

**Published:** 2016-11-23

**Authors:** Barbara Strasser, Daniela Geiger, Markus Schauer, Johanna M. Gostner, Hannes Gatterer, Martin Burtscher, Dietmar Fuchs

**Affiliations:** 1 Division of Medical Biochemistry, Biocenter, Medical University of Innsbruck, Innrain 80, 6020 Innsbruck, Austria; Johanna.Gostner@i-med.ac.at; 2 Division of Biological Chemistry, Biocenter, Medical University of Innsbruck, Innrain 80, 6020 Innsbruck, Austria; M.Sc.DanielaGeiger@gmail.com (D.G.); M.Schauer@hotmail.com (M.S.); Dietmar.Fuchs@i-med.ac.at (D.F.); 3 Department of Sport Science, Medical Section, University of Innsbruck, Fuerstenweg 189, 6020 Innsbruck, Austria; Hannes.Gatterer@uibk.ac.at (H.G.); Martin.Burtscher@uibk.ac.at (M.B.)

**Keywords:** intense exercise, kynurenine, tryptophan, probiotics, upper respiratory tract infections

## Abstract

Background: Prolonged intense exercise has been associated with transient suppression of immune function and an increased risk of infections. In this context, the catabolism of amino acid tryptophan via kynurenine may play an important role. The present study examined the effect of a probiotic supplement on the incidence of upper respiratory tract infections (URTI) and the metabolism of aromatic amino acids after exhaustive aerobic exercise in trained athletes during three months of winter training. Methods: Thirty-three highly trained individuals were randomly assigned to probiotic (PRO, *n* = 17) or placebo (PLA, *n* = 16) groups using double blind procedures, receiving either 1 × 10^10^ colony forming units (CFU) of a multi-species probiotic (*Bifidobacterium bifidum* W23, *Bifidobacterium lactis* W51, *Enterococcus faecium* W54, *Lactobacillus acidophilus* W22, *Lactobacillus brevis* W63, and *Lactococcus lactis* W58) or placebo once per day for 12 weeks. The serum concentrations of tryptophan, phenylalanine and their primary catabolites kynurenine and tyrosine, as well as the concentration of the immune activation marker neopterin were determined at baseline and after 12 weeks, both at rest and immediately after exercise. Participants completed a daily diary to identify any infectious symptoms. Results: After 12 weeks of treatment, post-exercise tryptophan levels were lowered by 11% (a significant change) in the PLA group compared to the concentrations measured before the intervention (*p* = 0.02), but remained unchanged in the PRO group. The ratio of subjects taking the placebo who experienced one or more URTI symptoms was increased 2.2-fold compared to those on probiotics (PLA 0.79, PRO 0.35; *p* = 0.02). Conclusion: Data indicate reduced exercise-induced tryptophan degradation rates in the PRO group. Daily supplementation with probiotics limited exercise-induced drops in tryptophan levels and reduced the incidence of URTI, however, did not benefit athletic performance.

## 1. Introduction

Numerous studies have shown that prolonged intense physical exercise is associated with a transient depression of immune function in athletes. While moderate exercise beneficially influences the immune system [1], a heavy schedule of training and competition can lead to immune impairment associated with an increased risk of upper respiratory tract infections (URTIs) due to altered immune function [2,3]. It has been suggested that exhaustive exercise creates a potential ‘open window’ of decreased host protection, during which viruses and bacteria can gain a foothold, increasing the risk of developing an infection [4]. During major competitions of 2–3 weeks duration, typically about 7% of athletes experience at least one episode of illness and about half of these are respiratory [5]. Exercise immunological studies reported that infection episodes were preceded by declines in immunoglobulin A (IgA) in saliva [6,7,8]. Furthermore, results suggest a possible mechanism for the increased incidence of infection during intensified training via modulation of type 1/type 2 T lymphocyte distributions [9].

Physical exercise and sports influence immunoregulatory circuits which, as a primary response, involve the production of forward regulatory cytokines is followed by counter-regulation leading to an immunosuppressed state [3,10,11]. Downstream biochemical events include changes in tryptophan (Trp) metabolism when T helper cell type 1 (Th1-type) cytokine interferon-γ (IFN-γ) is released and induces tryptophan-degrading enzyme indoleamine 2,3-dioxygenase (IDO-1). In turn, blood concentrations of Trp become reduced, leading to various potential consequences [12]. The essential amino acid Trp is not only a precursor of the serotonin biosynthesis pathway but is also the key element for the formation of the energy carrier and coenzyme nicotinamide-adenine-dinucleotide NAD and its reduced form NADH via the so-called kynurenine (Kyn) pathway [13,14]. Recently, exhaustive aerobic exercise in athletes was reported to significantly impact on Trp–Kyn metabolism [15]. Results indicate an involvement of IDO-1 activation in enhanced Trp catabolism and Kyn production following demanding exercise [15]. The close association of Trp metabolites with neuropsychopharmacologically relevant metabolites may have special consequences for athletes since it influences immunosurveillance and the development of infections as well training adherence because of disturbed neurotransmitter biochemistry [16].

Trp is also an important target for the gut and brain interaction [17]. In addition to its resorption from dietary components, the composition of gut bacteria—the microbiome—is of enormous importance in the regulation of Trp. Available data suggest a role for the gut microbiota in actually modulating Trp and hence having control over serotonin levels in the host [18]. Recently, an inverse correlation of serum levels of Trp, tyrosine, and phenylalanine with concentration of fecal calprotectin, a marker for gut leakiness, has been reported in patients suffering from Alzheimer’s disease, thus indicating a close relationship between the intestinal barrier function and aromatic amino acid concentration in the blood [19]. Furthermore, there is growing body of evidence indicating that the microbiota is sensitive to physiological changes associated with exercise [20,21]. For example, acute aerobic exercise reduces the expression of toll-like receptors (TLRs) in the monocyte cell-surface, contributing to post-exercise immunodepression, while over the long-term, a decrease in TLR expression may represent a beneficial effect because it decreases the inflammatory capacity of leukocytes, thus altering whole body chronic inflammation [22]. TLRs can activate dendritic cells, which are associated with the attenuation of immune activation and inflammation protection [20]. Notably, IDO-1 has been identified in mucosal Cluster of Differentiation 103 -expressing dendritic cells and has already been claimed to be a possible therapeutic target for gut disorders [23].

Dietary supplements containing probiotics can modify the population of the gut microflora and may provide a practical means of enhancing gut and systemic immune function, which was shown to be beneficial by reducing the infection frequency in sensible groups, e.g., elderly in group homes or children [24,25]. However, studies in these subject groups might not be reflective of athletes who have different gut microbiota [26]. Exercise and associated dietary extremes were shown to increase gut microbial diversity in comparison to sedentary people [27]. Some studies have established that probiotic intake can improve low-grade inflammation [28,29] and enhance resistance to URTI in athletes [30,31,32]. In a previous study, Lamprecht and colleagues found that adequate probiotic supplementation composed of six strains consisting of *Bifidobacterium bifidum* W23, *Bifidobacterium lactis* W51, *Enterococcus faecium* W54, *Lactobacillus acidophilus* W22, *Lactobacillus brevis* W63, and *Lactococcus lactis* W58 could improve redox hemostasis and low-grade inflammation in men under sustained exercise stress [29]. The mechanisms behind these observations have not been widely investigated but may include direct interaction with gut microbiota, interaction with mucosal immune system and modulation of lung macrophage and T cell functions [33]. For example, one study observed that the IFN-γ response (a potent stimulus for IDO-1) was moderately higher with probiotic treatment than with placebo, associated with a significant reduction in the number of days of respiratory illness symptoms in highly trained distance runners [30]. Since Trp availability is primarily regulated via the Kyn pathway, the catabolism of amino acid Trp via Kyn may play an important role on the risk of developing an infection.

The aim of the present study was to examine the effect of a probiotic supplement on the incidence of URTI and Trp metabolism after exhaustive aerobic exercise in trained athletes during three months of winter training We hypothesized that daily supplementation with probiotics is beneficial in reducing the incidence of URTI in athletes during training periods in winter and is associated with modulation of the Trp—Kyn metabolic pathways.

## 2. Materials and Methods

### 2.1. Subjects

Thirty-three healthy and trained volunteer athletes (mean age 26.7 years; average body mass index 22 kg/m^2^; average peak oxygen uptake 51.4 mL/kg/min) participated in this study that was conducted at the Department of Sport Science at the Leopold Franzens University of Innsbruck, Austria. Individuals were invited to participate if they were 20–35 years of age, non-smokers, had no previous history of muscle disorders and were free of heart, kidney, lung, neurologic, and psychiatric diseases. Athletes with a cardiorespiratory response and fitness of ≥150% of reference values during maximal exercise [34] were included. A questionnaire about medical history and previous training was filled out by each participant. In total, 33 individuals were enrolled with 29 participants (13 men 16 women) completing the study. Baseline characteristics of the subjects are presented in Table 1.

Subjects who met the inclusion criteria of the study were randomly assigned to the treatment or placebo group. The randomization code was held by a third party and handed over for statistical analyses after collection of all data. All of the participants were informed of the risks and potential discomforts associated with the investigation and signed a written consent to participate. The study was approved by the Board for Ethical Questions in Science Ethics at the Leopold Franzens University of Innsbruck according to the principles expressed in the Declaration of Helsinki.

### 2.2. Study Intervention

Subjects randomized to probiotics (PRO, *n* = 17) received boxes with sachets containing multi-species probiotics composed of six strains consisting of *Bifidobacterium bifidum* W23, *Bifidobacterium lactis* W51, *Enterococcus faecium* W54, *Lactobacillus acidophilus* W22, *Lactobacillus brevis* W63, and *Lactococcus lactis* W58 (Ecologic^®^ Performance, Winclove B.V., Amsterdam, The Netherlands). The total cell count was adjusted to 2.5 × 10^9^ colony forming units (CFU) per gram. The candidate strains were selected upon their survival in the gastrointestinal tract, activity, intestinal barrier function, and anti-inflammatory properties and were used in a previous study on immune health in athletes [29]. The matrix consisting of cornstarch, maltodextrin, vegetable protein, MgSO_4_, MnSO_4_ and KCl. Subjects were instructed to take 1 sachet of 4 g per day, which is equivalent to 1 × 10^10^ CFU/day, with 100–125 mL of plain water, one hour prior to breakfast and throughout the 12 weeks. Those subjects assigned to the placebo group (PLA, *n* = 16) received identical boxes and sachets with the same instructions for use.

### 2.3. Study Protocol

During the three-month intervention period (January 2015 to March 2015) subjects were asked to maintain their normal diet and to continue with their normal training programs. In addition, participants agreed to avoid taking medicine including anti-inflammatory drugs (e.g., aspirin, ibuprofen, voltaren) and antibiotics, additional probiotics and dietary supplements such as fish oil, vitamins (vitamin C, vitamin E) and minerals (selenium). Consumption of alcohol (>10 and 20 g for women and men, respectively, per day), or any fermented dairy products (e.g., yoghurt) was not permitted during this period. During the first visit to the laboratory, measures of participants’ weight and height were obtained using standardized methods and used to calculate body mass index (BMI, kg/m^2^). Prior to and at the end of the study, all subjects were tested for body fat (in percent of body weight), body cell mass (kg), and resting energy expenditure (kcal/day) using the bioelectrical impedance analysis (BIA) method (BIA-2000-M, Data Input, Pöcking, Germany). Prior to the first blood draw and after 12 weeks of supplementation, participants were asked to complete a three-day food record to evaluate energy and nutrient intake. Diet records were analyzed for total calories, protein, carbohydrate, fat, alcohol, and water intake using “nut.s science” nutritional software (dato Denkwerkzeuge, Vienna, Austria). Weekly training (modality, frequency, intensity, volume) and illness (URTI symptoms and gastrointestinal GI complaints symptoms) logs were kept.

The illness symptoms listed on the self-constructed questionnaire, modified according to Gleeson et al. (2011) [31] were sore throat, runny nose, cough, fever, and weakness. Subjects were asked to rate the severity of their symptoms (very light, light, moderate, severe, very severe). The GI discomfort symptoms listed on the questionnaire were abdominal pain, diarrhea, loss of appetite, vomiting, and others. The incidence score relates to the number of participants who reported symptoms in each arm of the study. One or more symptoms on at least two consecutive days were defined as an episode of illness. Symptoms with an interval of only one day were counted as the same episode.

### 2.4. Exercise Tests

In the morning of the exercise test a standardized breakfast was provided 2 h prior to strenuous exercise tests (379 kcal; 88 energy percent carbohydrates, 11 energy percent proteins, and 1 energy percent fat). The composition of this standardized breakfast is shown in Table 2.

For eligibility testing all subjects performed an incremental cycle ergometer exercise test until exhaustion. Cycle ergometry was performed on an electronically braked ergometer (Ergometrics 900, Ergoline, Germany) and started at a workload of 50/75 W (women/men) for 5 min (warm up) with a following increase in workload of 25 W per minute until exhaustion. Exhaustion was defined as the state when the pedaling rate dropped below 60 rpm. Heart rate and ventilatory parameters were monitored continuously (Oxycon mobile, Jaeger, Germany). Peak power output (P_max_) was defined as the last completed workload rate plus the fraction of time spent in the final uncompleted work rate multiplied by 25 W [35]. Peak oxygen uptake (VO_2max_) was defined as the highest 30-s average during the test.

After a 20 min resting period, athletes performed a 20-min maximal time-trial on a cycle ergometer (RBM Cyclus 2, Leipzig, Germany) as described by Faulhaber and colleagues [35]. Briefly, the cycle ergometer was shifted to a fixed pedal force in which power output was dependent on the pedaling rate. Pedal force for each participant was set so that pedaling at 100 rpm would produce about 70% (rounded to 5 W) of peak power output, which was determined by the incremental cycle ergometry. During the test, cyclists were strongly encouraged to choose a maximal pedaling rate that could be maintained for the respective test duration. The main outcome measurement was mean power output during the 20-min test, which was automatically calculated by the software of the ergometer. The participants were allowed to drink water ad libitum. Three months later this procedure was repeated on the same cycle ergometer and with the same investigator.

### 2.5. Blood Measurements

We conducted blood collections from the participants in the supine position from a medial cubital vein at baseline and after 12 weeks at rest and within 5 min after exercise (four blood draws per study participant). After centrifugation for 10 min cells were removed and plasma samples were frozen at −20 °C until analysis. Serum concentrations of Trp and Kyn as well as concentrations of phenylalanine (Phe) and tyrosine (Tyr) were determined by high-performance liquid chromatography (HPLC), as previously described [36,37]. The ratios of Kyn/Trp and Phe/Tyr were calculated as indexes of Trp degradation and phenylalanine 4-hydroxylase (PAH) activity, respectively. Pro-inflammatory cascades were found to be associated with disturbed PAH activity [37]. Serum neopterin concentrations were measured by ELISA (BRAHMS Diagnostics, Hennigsdorf, Germany) following the manufacturer’s instructions [38].

### 2.6. Statistical Analysis

Per protocol analyses were performed using SPSS (IBM SPSS Statistics Version 22, IBM Corp., Armonk, NY, USA). Normality in the distribution of data was tested using the Kolmogorov-Smirnov’s test and Boxplots. In the case of Gaussian distribution, baseline characteristics, performance data, nutrient and biological markers were compared by unpaired Student’s *t*-test or Mann-Whitney-*U*-Test. Changes in variables during the study were analyzed by univariate analysis of variance (ANOVA) for parametric variables. The Wilcoxon-signed rank and Friedman test were applied to non-parametric data. Spearman’s rank correlation was used to assess the association between two variables. Partial eta-squared values were calculated to estimate the effect of any statistically significant differences found. Using the guidelines of Cohen [39], 0.01 = small effect, 0.06 = moderate effect, and 0.14 = large effect. A *p*-value of less than 0.05 (two-tailed) was considered to indicate statistical significance. Data are presented as mean values ± standard deviation (SD) or by mean values ± standard error of the mean (SEM).

Sample size calculation was based on changes in exercise-induced Trp levels [40] from baseline to the end of the 12-week intervention between the PRO group and the control. We estimated between 10 and 12 subjects per group—depending on SD and effect size—to reach a probability of error (alpha/2) of 5% and 80% power. Allowing for a drop-out rate of 30%, 16 subjects per group were recruited.

## 3. Results

### 3.1. Study Population

**Figure 1:**
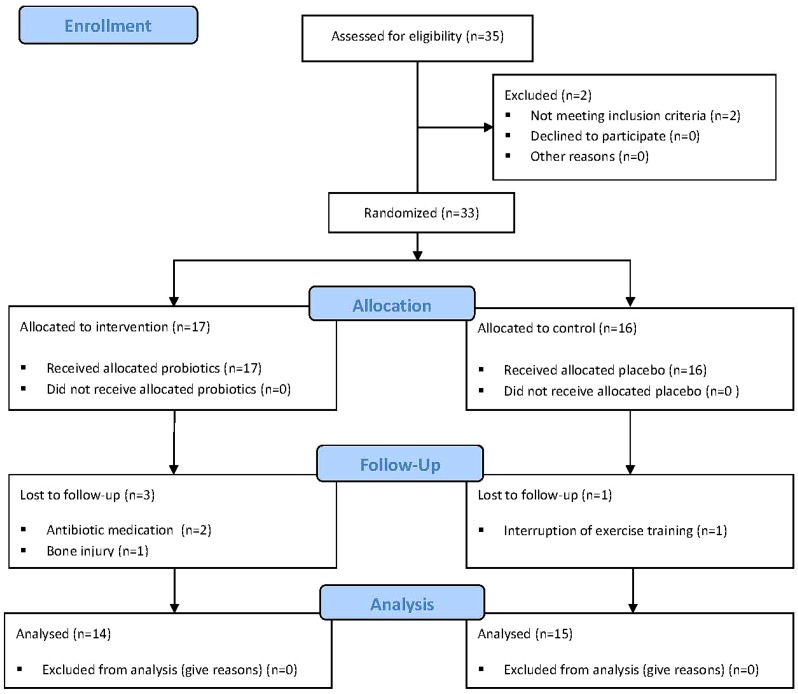
Flow of participations through each stage of the trial.

Twenty-nine of the 33 randomized subjects completed the full program and entered statistical analyses. Three withdrew because of injury or persistent illness with antibiotic medications, one because of a longer training interruption. Returned sachet count after the treatment period revealed a compliance rate >95% in both groups (97.6% in the probiotics group, 98.8% in the control group). The lowest level of compliance for a subject was 86.9%. A CONSORT (Consolidated Standards of Reporting Trials) diagram outlining participant recruitment is depicted Figure 1.

At baseline, a significant gender-dependent difference (females were overrepresented in the control group), VO_2max_ and Trp was observed between groups (*p* < 0.05). Females had a lower BMI, VO_2max_, and mean power output during the 20-min test (P_TT_) compared to male athletes, as Kyn levels were lower in females (*p* = 0.019). None of the other parameters were influenced by gender.

### 3.2. Training Loads

**Figure 2:**
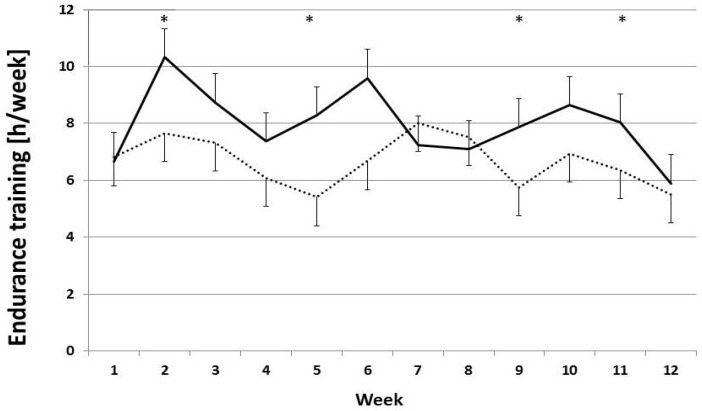
Training loads for endurance training (h/week) over the study period for the participants who completed the study. Graph shows mean ± standard error of the mean (SEM); * *p* < 0.05 (Mann-Whitney *U* test). Asterisks depict weeks with significant differences between PRO (—) and PLA (···) groups. PRO: probiotics-supplemented group; PLA: placebo group.

Analysis of training loads indicated that the weekly training of the aerobic system, mainly continuous endurance training at moderate intensity (60% to 80% VO_2max_), varied significantly between the groups over the 12-week treatment period (Figure 2). The means were significantly higher in the probiotics group as compared to the placebo group: 8.0 ± 2.3 and 6.6 ± 4.3 h per week endurance training, respectively (*U* = 2.597, *p* < 0.001).

### 3.3. Body Composition, Nutrition, and Performance

**Figure 3:**
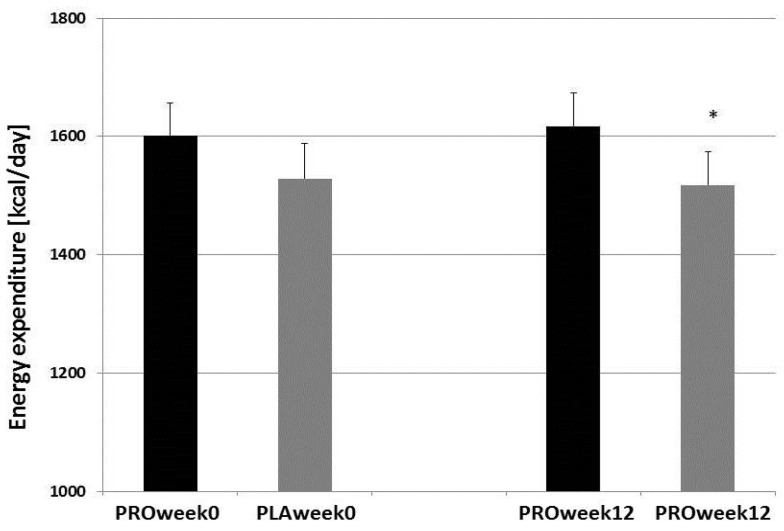
Resting energy expenditure (REE; (kcal/day)) in trained athletes before and after 12 weeks of treatment. PRO: probiotics-supplemented group (*n* = 14); PLA: placebo group (*n* = 15). Graph shows mean + SEM; * *p* < 0.05 (ANOVA).

After 12 weeks of treatment, there was no significant difference between probiotic supplementation groups and placebo groups in anthropometric characteristics, body composition, and food intake (*p* > 0.05). Performance (VO_2max_) remained unchanged over time and still differed significantly between groups in week 12 (*p* < 0.05). Resting energy expenditure (REE, kcal/day) was significantly different between groups after 12-weeks of the study (mean ± SEM: 1617 ± 57 kcal/day and 1518 ± 56 kcal/day for PRO and PLA, respectively; *p* < 0.05, η^2^ = 0.13; Figure 3).

### 3.4. Amino Acids

**Figure 4:**
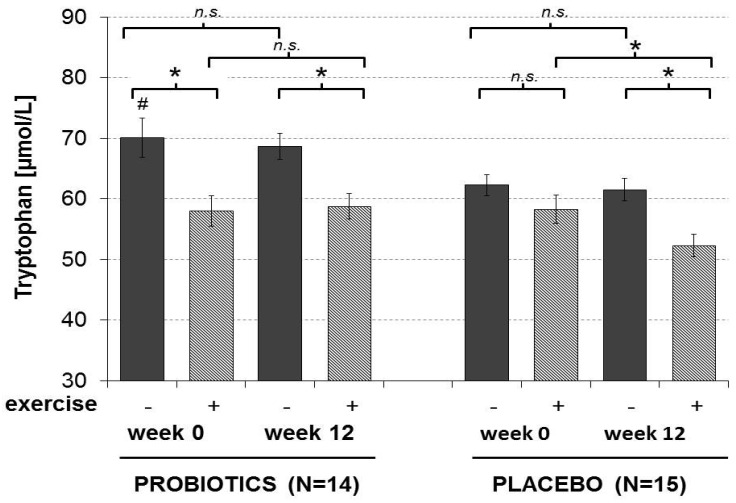
Tryptophan concentrations before and after exhaustive exercise in the probiotic (*n* = 14) and placebo (*n* = 15) group of trained athletes before and after 12 weeks of treatment (four blood draws per athlete). Graph shows mean ± SEM; * *p* < 0.05: Wilcoxon, # *p* < 0.05: week 0, before exercise placebo vs. probiotics: Mann-Whitney-*U*, n.s. = not statistically significant.

At the beginning of the study, exhaustive exercise induced a decrease in Trp levels in both the probiotic and the placebo group (Table 3). At the end of the experimental protocol, the exercise-induced Trp shift was comparable to the shift in week 0 in subjects who ingested probiotics but was more pronounced in in the placebo group (approximately 10% lower than in week 0, *p* < 0.05) (Figure 4).

These data indicate reduced Trp degradation rates in subjects supplemented with probiotics, although this effect was not significant (*p* = 0.13, η^2^ = 0.08). It should be mentioned that baseline Trp concentrations were slightly but significantly lower in the placebo group compared to the probiotics group, most probably due do the different percentage of female athletes in the groups. In parallel to Trp decrease, Kyn/Trp and neopterin levels were increased after exercise in both study groups at both time points.

Further, at the beginning of the study, VO_2max_ correlated significantly with baseline concentrations of Trp (*rs* = 0.562, *p* = 0.001) and this relation remained significant after 12 weeks of treatment (*r* = 0.497, *p* = 0.006) but was no longer present after intense exercise.

Tyrosine levels significantly increased and Phe/Tyr significantly decreased with exhaustive exercise (*p* = 0.018 and *p* < 0.001, respectively), but there were no significant time-dependent differences between groups. Serum concentrations of Phe were not significantly affected, either by exercise or by supplementation (Table 3).

### 3.5. Immune System Biomarkers

Exhausting exercise was associated with a strong increase in neopterin levels up to +61% (*U* = 4.420, *p* < 0.001) and +63% of pre-exercise values (*U* = 4.660, *p* < 0.001), before and after 12 weeks of treatment, respectively, with no significant differences between and within groups over time. However, this increase was significantly influenced by endurance training volume with a strong inverse correlation between the athletes’ training status and the concentrations of neopterin at exhaustion (*rs* = −0.502, *p* < 0.01).

Kyn concentrations were slightly increased with exercise by 7% (*U* = 2.671, *p* < 0.01) before and by 3% (*U* = 0.923, n.s.) after 12 weeks of intervention, contributing to the elevation of the Kyn/Trp ratios by 22% (*U* = 4.544, *p* < 0.001) and by 21% (*U* = 4.433, *p* < 0.001), respectively. Exercise induced a change in Kyn levels with time (∆Kyn), with a significant decline being overserved in the PLA group (*p* = 0.04), whereas an increase was seen in the PRO group, but this effect was not significant between groups (*p* = 0.05, η^2^ = 0.13). At baseline, neopterin and Kyn/Trp ratios correlated significantly (*rs* = 0.490, *p* < 0.01), with the association even becoming slightly stronger upon exercise (*rs* = 0.512, *p* < 0.01). After 12 weeks there was no longer a significant relationship between pre-exercise neopterin and Kyn/Trp levels (*rs* = 0.280, n.s.), but it became again significant after exercise (*rs* = 0.583, *p* = 0.001). At the same time, higher neopterin levels correlated with lower Trp concentrations (*rs* = −0.384, *p* < 0.05).

### 3.6. Infection Incidence

**Figure 5:**
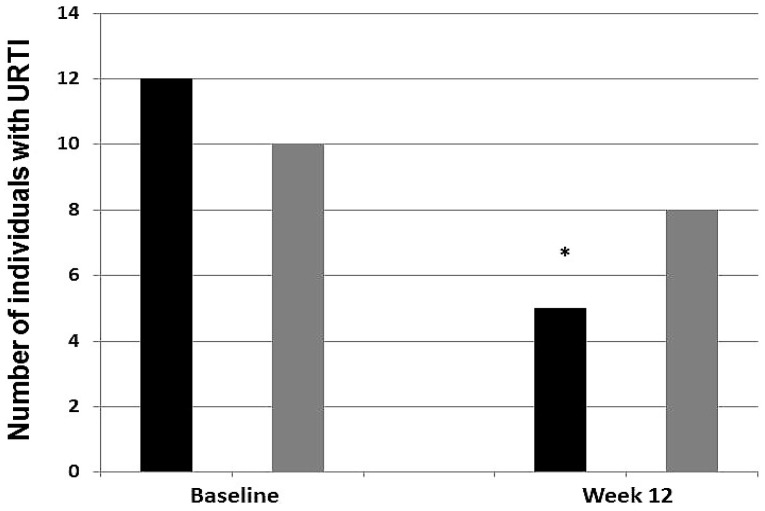
Incidence of upper respiratory tract infections (URTIs) in trained athletes before and after 12 weeks of treatment. The share of subjects on placebo (gray columns, 0.79) who experienced 1 or more URTI symptoms was 2.2-fold greater than those on probiotics (black columns, 0.35; * *p* = 0.016).

Only one participant on the placebo experienced GI-discomfort symptoms during the study period. Analysis of the URTI-symptom questionnaires indicated that 55% (16 subjects) of the cohort experienced an URTI episode during the 12-week study period. Thirteen subjects did not experience any URTI episode during the study period. Before supplementation, 10 subjects on placebo and 12 subjects on probiotics experienced one or more URTI symptoms over the prior three months. After 12 weeks of treatment, 11 subjects on placebo and 5 subjects on probiotics experienced one or more URTI symptoms during the study period (Figure 5). The proportion of subjects who experienced one or more URTI symptoms during the study period was 2.2-fold higher in the placebo group than in the probiotics group (PLA 0.79, PRO 0.35; *p* = 0.016).

Individuals who developed URTI had higher degradation rates of Trp before exercise compared to those without URTI (Table 4). Additionally, a running nose, but not cough was associated with higher Kyn/Trp ratios compared to those individuals without such symptoms.

## 4. Discussion

This study illustrates a significant influence of probiotic supplementation on athletes who performed intense exercise. On the one hand, increased training load was measured and on the other hand, the rate of infectious complications was markedly reduced. However, whether this is based on the actual probiotic supplementation or due to other cofounding factors (baseline fitness, gender) is currently unknown. In addition, some of these influences appeared to be connected with alterations in Trp metabolism, e.g., Trp breakdown rates at the end of the study were significantly higher in individuals who developed infections as compared to those who did not. However, it was not determined whether higher Kyn/Trp ratios were observed, particularly in those individuals who experienced an infection close to the end of the study and it still remains to be elucidated whether there is a more direct association between probiotic supplementation and reduced Trp breakdown. Alternatively, different training loads between groups may have affected Trp metabolism, rather than the actions of the probiotic, since regular endurance exercise causes adaptations in Kyn metabolism [41].

### 4.1. Training Adherence

Supplementation with probiotics was associated with higher training loads vs. placebo. One explanation for these findings could be that probiotics may enable better performance capabilities and training adherence when the risk of URTI development is reduced, as individuals with fewer episodes of infections such as common colds and runny noses are able to train more often and harder than others. However, it could also be possible that existing URTI symptoms influenced training performance to a lesser extent in athletes on probiotics [31]. In any case, performance was not increased even with the higher training load in the probiotics group as compared to the placebo group, even if the training load was indeed an effect of the supplementation.

A potential role of Trp metabolism could be of relevance for the effects of probiotics on training adherence because individuals on probiotics showed higher serum Trp levels than those without such supplements. Higher serum Trp levels may improve the Trp transport into brain and support serotonin metabolism, which can influence an individual’s sensation of fatigue and thus potentially affect training adherence and performance [42]. Interestingly, VO_2max_ correlated with pre-exercise Trp levels supporting a role of Trp metabolism in training performance. It could further relate to the recent findings of Kyn metabolism in skeletal muscle mediating resilience to stress-induced depression with endurance training, whereas less energetically demanding exercise protocols, such as high-force eccentric exercise, did not lead to adaptations in Kyn metabolism [41,43].

### 4.2. Tryptophan and the Gut Microbiome

Post exercise serum Trp levels declined but this was only true in the placebo group whereas serum Trp levels did not change but remained stable in individuals supplemented with probiotics. This difference could be due to an effect of probiotics on the microbiome composition in the gut, which may affect downstream immunoregulatory pathways. Alterations in the gut milieu influence Trp metabolism and the absorption and availability of the essential amino acids [17]. In addition, the altered composition of the microbiome may increase the biosynthesis of Trp by specific bacteria. Research in rats has shown that administration of the probiotic *Bifidobacteria infantis* attenuated pro-inflammatory immune responses following mitogen stimulation and, furthermore, there was a marked increase in plasma concentrations of Trp in the *Bifidobacteria*-treated rats when compared to controls [44], suggesting that bacteria can improve the available serotonin pool and ultimately elicit communication between the gut and the brain via serotonin [17,18]. In the present study, probiotics were able to selectively modulate Trp concentrations since no influence on the metabolism of Phe, another essential amino acid, was observed. Interestingly, no detectable effect of supplementation was found on concentrations of immune system biomarker neopterin and also Kyn/Trp ratios were not modulated. Further studies will be necessary to address these open questions.

### 4.3. Probiotics to Prevent URTIs

Some well-controlled studies in athletes have shown that daily probiotic ingestion results in fewer days of respiratory illness and lower severity of URTI symptoms [30,31,32]. A meta-analysis using data from both athlete and non-athlete studies concluded that there is a likely benefit of reducing URTI incidence [45]. The likely mechanisms of action for probiotics include direct interaction with the gut microbiota, interaction with the mucosal immune system and immune signaling to a variety of organs and systems [46]. A recent report by He and colleagues noted gender differences in the number and duration of respiratory-tract illness symptoms in endurance athletes during a winter training period indicating that females may be more susceptible to URTI than their male counterparts [5]. Furthermore, supplementation with *Lactobacillus fermentum* was associated with a reduction of the symptoms in clinical indices of URTI at high training loads in well-trained male cyclists but not in females, for whom there was some evidence of an increase in symptoms [47]. Thus, females may benefit from higher doses of probiotics. In the present study, females were overrepresented in the control group who experienced more than double the URTI symptoms, accompanied by higher Trp breakdown rates compared to those on probiotics. However, gender is not the basis for the observed effect on URTI incidence in this study. The interaction between gender and URTI was not statistically significant in either group before and after 12 weeks of treatment (Table 5). It remains unclear whether these findings are related to the influence of gender on Trp catabolism and further work is required to address this issue.

Taken together, probiotic supplements on a daily basis enhance resistance to URTI in athletes and offer thus a potential intervention strategy during heavy exercise training periods, especially in the winter months. A prerequisite for robust immune function during intense exercise is, however, to avoid a long-term energy deficit, deficiencies of macronutrients and essential micronutrients, and to ingest carbohydrate during exercise [48].

### 4.4. Study Strengths and Limitations

This study has several strengths and limitations. A major strength was the randomized controlled, double-blinded, placebo-controlled study design and the use of an objective and standardized test for assessment of peak oxygen uptake and peak power output. The study was conducted with a multi-species probiotic consisting of *Bifidobacterium bifidum* W23, *Bifidobacterium lactis* W51, *Enterococcus faecium* W54, *Lactobacillus acidophilus* W22, *Lactobacillus brevis* W63, and *Lactococcus lactis* W58 at a total dose of 1 billion CFU. Total probiotic cell count varied between 11 × 10^9^ CFU/g at the beginning of the study and 7 × 10^9^ CFU/g at the end of the study. The individual amount of each strain is unknown. Returned sachet count after the treatment period revealed a high level of compliance in both groups (>95%). The 80% cutoff has been used for a majority of studies on medication adherence, especially for cardiovascular medications, since adherence based on this cutoff point has been associated with both intermediate and strong outcomes [49]. Although the 80% cutoff appears reasonable, the optimal level of adherence for dietary supplements may be higher than current cutoffs (e.g., 80% to 100%). Limitations of the study are the relatively small sample size and significant differences in gender composition of the subpopulations, which may have contributed to differences in illness symptoms and physiological parameters, e.g., VO_2max_ and weekly training logs between males and females. We did not randomize by body weight, performance, or any other variable that might have given us a better chance to catch differences in outcomes since women—overrepresented in the control group—generally show a lower VO_2max_. Indeed, West et al. (2011) showed gender difference with probiotic supplementation in athletes with a significant reduction in respiratory infections (duration and severity) in males, but no effect in females [47]. Therefore, we analyzed the influence of gender on URTI. No significant effect of gender was observed for either group, before or after 12 weeks of treatment. However, Trp metabolism can be influenced to some extend by gender differences [50]. This aspect is mainly relevant for the results obtained at the baseline when Kyn levels were found to differ between groups. Another limitation of the study is that we were not able to calculate the severity of illness symptoms because of the high number of no replies. Furthermore, infections were only symptomatically monitored, but not serologically proven. It is assumed that common pathogens either of bacterial or viral origin have been involved in and could contribute to alterations of, e.g., Trp metabolism, which, because of its immunoregulatory influence, could increase the risk of such infections. Longitudinal research will be needed to clarify causal ordering.

## 5. Conclusions and Future Research

Daily supplementation with probiotics was found to be associated with a lower frequency of URTIs in athletes who underwent endurance training and seems to be beneficial in increasing training efficacy during training periods, however, no benefits to athletic performance were observed. Some of these effects appeared to be connected with alterations in Trp metabolism. Still, these findings are of a preliminary nature and warrant further investigation into the precise mechanisms involved. In addition, more research is required to clarify issues of strains, dose–response, mechanisms and best practice models for probiotic implementation in various sports disciplines. It should be further investigated as to whether regular exercise per se affects human microbiota characteristics, for how long and how much exercise is needed.

## Figures and Tables

**Table 1:** Baseline characteristics, nutrition and performance data of the participants.

Variable	Unit	Probiotics (*n* = 14) Mean ± SD	Placebo (*n* = 15) Mean ± SD
Gender	male/female	8/6	5/10
Age	year	25.7 ± 3.5	26.6 ± 3.5
BMI	kg/m^2^	22.2 ± 1.5	21.2 ± 2.7
Weight	kg	67.4 ± 9.6	62.9 ± 11.1
Body cell mass	kg	31.2 ± 6.6	28.7 ± 7.4
Total body fat	%	20.1 ± 5.7	19.5 ± 4.4
VO_2max_	mL/kg/min	55.1 ± 6.4	47.5 ± 7.1 **
P_max_	watt	325 ± 54.2	274 ± 51.6 *
P_rel_	watt/kg	4.8 ± 0.3	4.3 ± 0.4 **
P_TT_	watt	222 ± 41.9	181 ± 38.3 *
Energy intake	kcal/day	2821 ± 1374	2840 ± 1161
REE	kcal/day	1602 ± 206	1519 ± 2031
Protein	%	14.9 ± 3.3	15.0 ± 3.5
Carbohydrates	%	49.5 ± 12.4	49.3 ± 12.7
Fat	%	32.5 ± 10.8	33.0 ± 12.1
Fibers	g	33.0 ± 10.1	32.0 ± 14.2
Alcohol	g	11.1 ± 10.7	9.4 ± 9.5
Water	L	3.38 ± 0.58	3.37 ± 0.84

Values are means ± SD; Significant difference between the groups: * *p* < 0.05; ** *p* < 0.01; BMI: body mass index; VO_2max_ = peak oxygen uptake; P_max_ = peak power output; P_rel_ = peak power output related to body weight; P_TT_ = Time-trial power output; REE = resting energy expenditure.

**Table 2:** Composition of the standardized breakfast 2 h prior to strenuous exercise tests.

Food	Energy (kcal)	Protein (g)	Carbohydrates (g)	Fat (g)
2 wheat rolls 100 g	260	8.70	52.7	0.90
Marmalade/jam 50 g	114	0.30	28.0	0.00
250 mL tea	5	0.75	0.25	0.25
250 mL water	-	-	-	-
Total	379	9.75	80.95	1.15
Meal energy (%)		11	88	1

**Table 3:** Amino acids and immune biomarkers in 29 athletes before and after 12 weeks of treatment either supplemented with probiotics or placebo measured before (PRE) and after exercise (POST).

**Probiotics (*n* = 14)**	**Baseline PRE**	**Baseline POST**	**Week 12 PRE**	**Week 12 POST**
Tryptophan (µmol/L)	70.07 ± 3.20 ^a,e^	57.99 ± 2.47 ^b^	68.64 ± 2.12 ^c,k^	58.76 ± 2.11 ^d^
Kynurenine (µmol/L)	1.98 ± 0.11	1.97 ± 0.07	1.83 ± 0.10	1.92 ± 0.11
Kyn/Trp (µmol/mmol)	28.35 ± 1.16 ^f^	34.50 ± 1.46	26.94 ± 1.51 ^l^	33.32 ± 2.19
Neopterin (nmol/L)	5.19 ± 0.23 ^g^	8.43 ± 1.00	4.92 ± 0.31 ^m^	7.74 ± 0.86
Tyrosine (µmol/L)	138.58 ± 29.96 ^h^	145.06 ± 6.23	147.25 ± 24.01 ^n^	149.22 ± 5.60
Phenylalanine (µmol/L)	69.59 ± 8.27 ^i^	68.72 ± 2.06	72.16 ± 1.99 ^o^	71.76 ± 1.90
Phe/Tyr (mol/mol)	0.52 ± 0.08 ^j^	0.48 ± 0.01	0.50 ± 0.03 ^p^	0.49 ± 0.02
**Placebo (*n* = 15)**	**Baseline PRE**	**Baseline POST**	**Week 12 PRE**	**Week 12 POST**
Tryptophan (µmol/L)	62.27 ± 1.72	58.27 ± 2.37	61.50 ± 1.84	52.26 ± 1.86
Kynurenine (µmol/L)	1.77 ± 0.13	2.02 ± 0.07	1.75 ± 0.08	1.77 ± 0.09
Kyn/Trp (µmol/mmol)	28.38 ± 1.81	34.49 ± 2.17	28.49 ± 1.03	34.03 ± 1.51
Neopterin (nmol/L)	6.63 ± 0.95	10.48 ± 1.56	5.65 ± 0.70	9.55 ± 2.06
Tyrosine (µmol/L)	131.15 ± 5.28	137.40 ± 5.42	126.41 ± 6.29	129.28 ± 5.76
Phenylalanine (µmol/L)	69.23 ± 2.45	68.55 ± 1.87	72.53 ± 1.67	70.09 ± 2.71
Phe/Tyr (mol/mol)	0.53 ± 0.02	0.50 ± 0.02	0.59 ± 0.03	0.55 ± 0.02

Values are means ± SEM. ^a^ *U* = 2.095, *p* = 0.036 (baseline PRE placebo vs. probiotics), ^b^ *U* = 0.284, *p* = 0.777 (baseline POST placebo vs. probiotics), ^c^ *U* = 2.706, *p* = 0.007 (week 12 PRE placebo vs. probiotics), ^d^ *U* = 2.139, *p* = 0.032 (week 12 POST placebo vs. probiotics), ^e^ *U* = 3.384, *p* = 0.001 (all athletes baseline PRE vs. POST), ^f^ *U* = 4.660, *p* < 0.001 (all athletes baseline PRE vs. POST), ^g^ *U* = 4.420, *p* < 0.001 (all athletes baseline PRE vs. POST), ^h^ *U* = 2.011, *p* = 0.044 (all athletes week 12 PRE vs. POST), ^i^ *U* = 0.270, *p* = 0.787 (all athletes week 12 PRE vs. post), ^j^ *U* = 3.357, *p* = 0.001 (all athletes week 12 PRE vs. post), ^k^ *U* = 4.703, *p* < 0.001 (all athletes week 12 PRE vs. POST), ^l^ *U* = 4.433, *p* < 0.001 (all athletes week 12 PRE vs. post), ^m^ *U* = 4.544, *p* < 0.001 (all athletes week 12 PRE vs. post), ^n^ *U* = 0.443, *p* = 0.658 (all athletes week 12 PRE vs. post), ^o^ *U* = 0.660, *p* = 0.510 (all athletes week 12 PRE vs. post), ^p^ *U* = 1.208, *p* = 0.227 (all athletes week 12 PRE vs. post).

**Table 4:** Association between upper respiratory tract infection (URTI) incidence at week 12 and degree of tryptophan breakdown as indicated by Kyn/Trp (mean ± SEM). Bold text indicates a statistically significant correlation with a *p*-value less than 0.05.

URTI	Baseline PRE	Baseline POST	Week 12 PRE	Week 12 POST
yes	28.9 ± 1.7	34.9 ± 6.0	31.1 ± 5.1	38.9 ± 7.3
no	28.2 ± 6.5	34.4 ± 7.2	26.7 ± 4.3	32.0 ± 6.0
*U*/*p*-value	0.535/0.592	0.102/0.919	**2.039/0.041**	**2.090/0.037**

**Table 5:** Interaction between gender and illness symptoms in 29 athletes before and after 12 weeks of treatment either supplemented with probiotics (PRO) or placebo (PLA).

Illness Symptoms		Baseline *U*	*p*	Week 12 *U*	*p*
URTI	PRO	0.212	0.832	0.155	0.877
	PLA	0.748	0.454	0.399	0.690
Runny nose	PRO	0.212	0.832	0.329	0.742
	PLA	0	1	1.080	0.280
Cough	PRO	0.931	0.352	0.362	0.717
	PLA	1.497	0.134	1.497	0.134
Sore throat	PRO	1.041	0.298	0.866	0.386
	PLA	0.374	0.708	1.497	0.134
Fever	PRO	0.823	0.411	0	1
	PLA	0	1	0	1
Weakness	PRO	0.866	0.386	0	1
	PLA	0.519	0.604	0.519	0.604
